# Sexual imprinting leads to speciation in locally adapted populations

**DOI:** 10.1002/ece3.9479

**Published:** 2022-11-08

**Authors:** Richard M. Sibly, Robert N. Curnow

**Affiliations:** ^1^ School of Biological Sciences University of Reading Reading UK; ^2^ Department of Mathematics and Statistics University of Reading Reading UK

**Keywords:** assortative mating, genetic polymorphism, migration selection balance, parapatric speciation, sexual imprinting

## Abstract

Sexual imprinting is widespread in birds and other species but its existence requires explanation. Our results suggest that sexual imprinting leads to speciation in locally‐adapted populations if a neutral mating cue—e.g., novel plumage coloration—arises through mutation. Importantly, the mating cue locus is not linked to adaptation loci. Local adaptation is a necessary precursor to speciation and occurs when evolution results in stable genetic polymorphisms with one allele predominating in some areas while others predominate elsewhere. Here we use a deterministic two‐niche population genetic model to map the set of migration and selection rates for which polymorphic evolutionary outcomes, i.e., local adaptations, can occur. Approximate equations for the boundaries of the set of polymorphic evolutionary outcomes were derived by Bulmer (*American Naturalist*, 106, 254, 1972), but our results, obtained by deterministic simulation of the evolutionary process, show that one of Bulmer's equations is inaccurate except when the level of dominance is 0.5, and fails if one of the alleles is dominant. Having an accurate map of the set of migration and selection rates for which polymorphic evolutionary outcomes can occur, we then show using the model of Sibly et al. (*Ecology and Evolution*, 9, 13506, 2019) that local adaptation in all analyzed cases leads to speciation if a new neutral mating cue arises by mutation. We finish by considering how genome sequencing makes possible testing our model and its predictions.

## Graphical abstract

We show that given sexual imprinting and local adaptation, a mutation giving males a new mating cue such as a new plumage colour will rapidly spread through a population and in so doing lead to speciation. This could explain the diversity of plumage colours seen in birds and cichlids, for example. This idea contrasts with the widely held view that speciation in birds generally follows a period of allopatry.

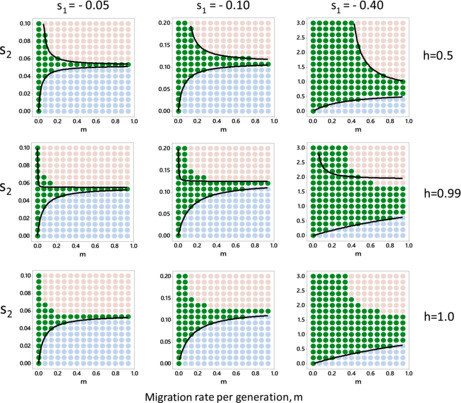

## 1 INTRODUCTION

Sexual imprinting is a process whereby individuals choose mates that resemble other individuals, usually one of their parents. Sexual imprinting seems to be a general feature of birds, shown to exist in over 100 species belonging to 15 different orders, and in both sexes (ten Cate & Vos, 1999). ten Cate and Vos (1999) noted that theoretical models for evolutionary processes had generally ignored the fact that mate preferences in many birds are acquired by sexual imprinting. Sexual imprinting has been found in other taxa too, including mammals, fish (cichlids and stickleback) and frogs (Verzijden et al., 2012; Yang et al., 2019). Sexual imprinting results in phenotype matching, such that individuals mate with others who resemble themselves. The existence and maintenance of sexual imprinting requires explanation. Here we suggest that it always leads to speciation in locally‐adapted populations if a neutral mating cue—e.g., novel plumage coloration—arises through mutation.

Speciation in birds is currently considered to occur after a period of allopatry, during which a population becomes split into geographically isolated subpopulations which remain separate and diverge over thousands of generations, and eventually can no longer interbreed (Tobias et al., 2020). One alternative to allopatric speciation is parapatric speciation in which speciation occurs within populations that are exchanging genes. Although currently there is only limited evidence for parapatric speciation, here we argue that its potential importance should be reconsidered because of the ease with which it occurs in sexually‐imprinting species in which there are local adaptations. How mate‐choice mechanisms affect speciation in the presence of local adaptation to differing ecological niches has been extensively studied. Existing studies derive ultimately from the insights of Felsenstein (1981); see also Butlin et al. (2021). Felsenstein (1981) mainly considered haploid models in which the alleles of some loci confer adaptation to one or other of two habitats, while another linked locus, which is not under direct selection, causes assortative mating (Butlin et al. (2021) term this a “two‐allele mechanism”). Felsenstein showed that in the presence of migration some direct selection is necessary for a population to evolve towards speciation. Reviews of the subsequent literature can be found in Butlin et al. (2012), Butlin and Smadja (2018), Gavrilets (2004, 2014), Kirkpatrick and Ravigne (2002), Kopp et al. (2018), Smadja and Butlin (2011), Yeh and Servedio (2015). These studies show that the evolution of prezygotic isolation is favored because it reduces the production of maladaptive genotypes, a process known as reinforcement (Butlin & Smadja, 2018). In the simplest case, the mating trait—the trait used in choosing a mate—is a magic trait, meaning it is itself under natural selection. Magic‐trait mate choice is known to produce speciation in the presence of ecological divergence between niches (Kopp et al., 2018; van Doorn et al., 2009). The effect of sexual imprinting on speciation in the presence of local adaptation, the subject of the present paper, has received less attention.

For sexual imprinting to result in evolutionary change, variation in the mating trait is necessary. The source of such variation has been variously posited. Verzijden et al. (2005) assumed variation in the mating trait was provided by frequency‐dependent natural selection acting on the mating trait: traits at frequencies above ½ were assumed to be selected against. With this assumption they found that the inheritance mechanism of mate choice can have a large effect on the ease of sympatric speciation: when females imprint on their mothers speciation can occur fairly easily, but when females imprint on their fathers or imprint obliquely, speciation becomes considerably less likely. Yeh and Servedio (2015) assumed the existence of initial variation in a mating trait between two populations experiencing gene flow, and analyzed learning of self‐referent phenotype matching—a proxy for some types of sexual imprinting. In this case, they showed that divergence between populations can be more easily maintained if traits are learnt from father than from mother. Yang et al. (2019) assumed the existence of variation in skin color in poison frogs and showed that stable color polymorphism can be a result of both sexes imprinting on the color of their mothers, if this drives mate choice by females and aggression in males. Here we study the case that initial variation in the mating trait is the result of a mutation in the mating trait in populations that already differ in an ecological trait as a result of local adaptation. This case was studied by Sibly et al. (2019), who used a simple diploid population genetic model to show how phenotypic matching based on neutral mating cues enables speciation in locally adapted populations. Crucially, the mating cues occur at a locus not linked to the adaptation locus. Sibly et al.'s (2019) analysis revealed that in the presence of local adaptation a mating trait can rapidly enable speciation given a suitable mechanism for phenotype matching. Only a limited number of cases of local adaptation were examined, however, so this raises the question, studied here, as to whether speciation is favored wherever local adaptation exists.

Our paper is in two sections. We begin with a systematic study to map the migration rates and selection strengths that allow the existence of local adaptation (i.e., migration‐selection balance). Then we extend the model of Sibly et al. (2019) to include intermediate levels of dominance, and apply this extended model to our map of the set of migration and selection rates for which local adaptations can occur. This shows that local adaptations always lead to speciation if a new neutral mating trait arises by mutation. We finish by considering how genome sequencing makes possible testing of our results.

## 2 SECTION 1. WHAT COMBINATIONS OF MIGRATION RATE AND SELECTION CAN PRODUCE LOCAL ADAPTATIONS?

Theoretical interest in quantifying the conditions under which local ecological adaptations arise goes back to Wright (1931) and Dobzhansky (1937). Guided by the results of experimental work on genetic variation in fruit flies (*Drosophila* species), Dobzhansky (1937) proposed that the persistence of genetic variation was owing to the action of balancing selection, in which heterozygotes are more fit than either homozygote, and Levene (1955) established the conditions under which such polymorphisms are stable in an influential single‐locus diploid two‐patch model. In the following decades balancing selection, and spatially varying selection in particular, became popular explanations for the maintenance of genetic variation, and interest has continued with the advent of genomic analyses (Gloss & Whiteman, 2016; Hedrick, 2006). Balancing selection is however not the only possible genetic mechanism, and models of dominance and semidominance, also deserve attention.

In the two‐patch model of Levene (1955), selection and population regulation occur within patches, individuals congregate for breeding into a single population within which they mate at random, and stable polymorphism is maintained by balancing selection. Our interest is in models like Levene's except that heterozygotes are not more fit than either homozygote, and individuals do not congregate for breeding into a single population. Instead they breed within patches after some have migrated between patches. This situation was analyzed in an infinite‐population model by Bulmer (1972), who derived recurrence equations relating gene frequencies in one generation to their frequencies in the next, and investigated the stability of resulting equilibria by supposing that perturbations about equilibria are small enough that their squares and higher powers can be ignored. This allowed him to derive equations for the boundaries of the set of polymorphic evolutionary outcomes, here termed the *polymorphism set*, whatever the selective advantages and migration rates between two niches. Bulmer's analysis applies whatever the level of dominance and selective advantages in the two niches. Subsequent work has taken Bulmer's equations for the boundaries of the polymorphic set as a starting point, but has not moved beyond them for the case of infinite‐population models (see, e.g., Nagylaki, 2009; Tomasini & Peischl, 2018; Whitlock, 2015; Yeaman & Otto, 2011). Gavrilets (2004) showed that stable polymorphism (ecological adaptation) is always achieved in a two‐niche model in which heterozygotes have fitness half way between the two homozygotes and selection is assumed symmetrical, the advantage of one allele in one niche being balanced by an equal disadvantage in the other niche. A similar two‐niche model allowed one of the alleles to be dominant (Bolnick & Nosil, 2007), but again assumed symmetrical selection, and lacked analysis of the factors that allow the establishment of stable ecological polymorphisms, the subject of the present paper. In finite populations, drift is important, and its effects have been studied by Yeaman and Otto (2011). Yeaman and Otto (2011) used Kimura's approach (Kimura, 1962) based on diffusion equations to obtain approximate results showing how the polymorphic set is affected by drift in small populations. Individual‐based simulations then showed that these approximations provide accurate predictions across a wide range of parameter values. The presence of drift reduces the chance that beneficial mutations become established, and Tomasini and Peischl (2018) have derived an analytical approximation for the probability that new mutations escape genetic drift and become permanently established, for the case of weak positive selection in one of the niches. This allows them to establish the effects on establishment probabilities of migration rates, the strength of selection in each niche, and the niches' carrying capacities. Despite these advances Bulmer's (1972) remains the most general deterministic infinite‐population analysis of the factors that allow the establishment of stable ecological polymorphisms, and his equations provide the only available means of characterizing in terms of selective advantages and migration rates the boundaries of the set of polymorphic evolutionary outcomes, here termed the polymorphism set.

Here we use recursive application of (Bulmer's, 1972) recurrence equations to investigate the accuracy of Bulmer equations for the boundaries of the polymorphism set in a deterministic two‐niche single‐locus model. We find good agreement about one of the boundaries, and about the other when the level of dominance, h, equals 0.5, but not at h = 0.99 because of a failure of the linearity assumption in Bulmer's stability analysis. We show and explain why Bulmer's analysis fails to find one of the boundaries when h = 1 (dominance). Overall, however, the Bulmer boundaries calculated at h = 0.5 provide a fairly good approximation of the true boundaries at other levels of dominance, particularly when selection is weak.

### 2.1 Methods

**FIGURE 1:**
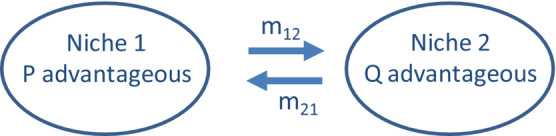
Conceptual overview of the model. For clarity, the niches are shown distinct, but in nature may be contiguous or overlap. m_12_ and m_21_ specify the proportion of individuals in one niche that migrate to the other each generation after viability selection and population regulation have occurred. When analyzing the model we suppose that Q is disadvantageous in niche 1 (i.e., s_1_, defined below, is negative) but advantageous in niche 2 (i.e., s_2_ is positive), while PP homozygotes have fitness 1 in both niches. There are no sex differences in fitnesses or migration rates.

The model of local adaptation analyzed here has one locus with two alleles P and Q in an environment consisting of two niches with some migration between niches prior to mating, as depicted in Figure 1. The locus determines ecological adaptation to one niche or the other. We assume a large population so that the dynamics are deterministic. Generations are discrete and individuals die after mating. The life histories occur in the following order. At the start of each generation individuals in each niche mate at random, and all mating individuals obtain the same number of offspring. The number of offspring of each genotype that survive in each niche is the product of its initial frequency and its fitness. Population regulation then returns population numbers to their initial values. Finally some individuals migrate between niches, as shown in Figure 1, leading to the start of the next generation.

#### 2.1.1 Bulmer's equations for the boundaries of the polymorphism set

We begin by rederiving Bulmer (1972) equations for the boundaries of the polymorphism set (see also Yeaman & Otto, 2011). This lets us see why Bulmer's analysis accurately identifies one boundary but not the other. Bulmer (1972) used transition matrices relating the frequencies of the alleles in the two niches in successive generations to derive equations for the boundaries separating regions where polymorphisms are stable from regions where one or other allele is bound to move to fixation. We prefer a notation in terms of levels of dominance rather than that used by Bulmer (1972), the relationship between the two notations is shown in Table 1.

**TABLE 1:** Assignment of fitnesses to genotypes by Bulmer (1972) and the present paper

Genotype	PP	PQ	QQ
Fitnesses in Bulmer (1972)	1 − x	1	1 − y
Fitnesses in our notation	1	1 + hs	1 + s
Frequency after mating	p^2^	2pq	q^2^

*Note*: In our scheme, h is the level of dominance of the Q allele. p and q are the frequencies of the P and Q alleles respectively; p + q = 1.

Parameter values in the two niches will be distinguished with subscript 1 and 2. To obtain the same fitness ratios of genotypes in Bulmer's scheme and ours we need:

(1) $$\frac{1}{\left(1-x_{i}\right)}=1+{\mathrm{hs}}_{i}$$

(2) $$1-y_{i}=\frac{\left(1+s_{i}\right)}{\left(1+{\mathrm{hs}}_{i}\right)}$$

where subscript i takes values 1 or 2 in niches 1 and 2 respectively.

In Bulmer's notation, the relative frequencies of P in the two niches after selection are:

(3a) $$p_{1}^{\prime }=\frac{\left(p_{1}-x_{1}p_{1}^{2}\right)}{\left(1-x_{1}p_{1}^{2}-y_{1}q_{1}^{2}\right)}$$

(3b) $$p_{2}^{\prime }=\frac{\left(p_{2}-x_{2}p_{2}^{2}\right)}{\left(1-x_{2}p_{2}^{2}-y_{1}q_{2}^{2}\right)}$$

The relative frequencies of P after migration are:

(4a) $$p_{1}^{\prime \prime }=\left(1-m_{12}\right)p_{1}^{\prime }+m_{21}p_{2}^{\prime }$$

(4b) $$p_{2}^{\prime \prime }=\left(1-m_{21}\right)p_{2}^{\prime }+m_{12}p_{1}^{\prime }$$

where p_1_' and p_2_' are obtained from equations 3.

Bulmer argued that stable intermediate equilibria in the two niches will occur if the equilibria at p = 0 and p = 1 are both unstable, so that, in either case, one allele is able to invade a population consisting entirely of the other. Ignoring quadratic terms in e_1_and e_2_, the equilibria at p = 0 would only be unstable if small perturbations p_1_ = e_1_ and p_2_ = e_2_ resulted in increased values of e_1_ and e_2_ in the next generation. From equations (3) and (4) this gives:

(5a) $$\frac{\left(1-m_{12}\right)e_{1}}{\left(1-x_{1}\right)}+\frac{m_{21}e_{2}}{\left(1-x_{2}\right)}>e_{1}$$

(5b) $$\frac{\left(1-m_{21}\right)e_{2}}{\left(1-x_{2}\right)}+\frac{m_{12}e_{1}}{\left(1-x_{1}\right)}>e_{2}$$

These inequalities give the conditions for equilibria at p = 0 to be unstable. The boundary between stability and instability occurs when the inequalities are replaced by equalities, so that the left‐hand and right‐hand sides of equations 5 are equal. Equations 5 can then be simplified to give the equation of the boundary as:

(6a) $$\frac{m_{12}}{x_{1}}+\frac{m_{21}}{x_{2}}=1.$$

This is the boundary at p = 0. The same reasoning as above gives the boundary at p = 1 as:

(6b) $$\frac{m_{12}}{y_{1}}+\frac{m_{21}}{y_{2}}=1.$$

Equations 6a and 6b are the equations Bulmer (1972) derived using the characteristic roots of transition matrices, for the boundaries of the regions where polymorphisms are stable. These equations can be expressed in our notation by substituting for x_1_, x_2_, y_1_ and y_2_ from Equations (1) and (2) to obtain:

(7a) $$s_{2}=\frac{m_{21}}{h\left(1-m_{21}-\frac{m_{12}\left(1+hs_{1}\right)}{hs_{1}}\right)}$$

(7b) $$s_{2}=\frac{m_{21}}{\left(h-1\right)\left(1-\frac{m_{12}\left(1+hs_{1}\right)}{\left(h-1\right)s_{1}}\right)-hm_{21}}$$

**FIGURE 2:**
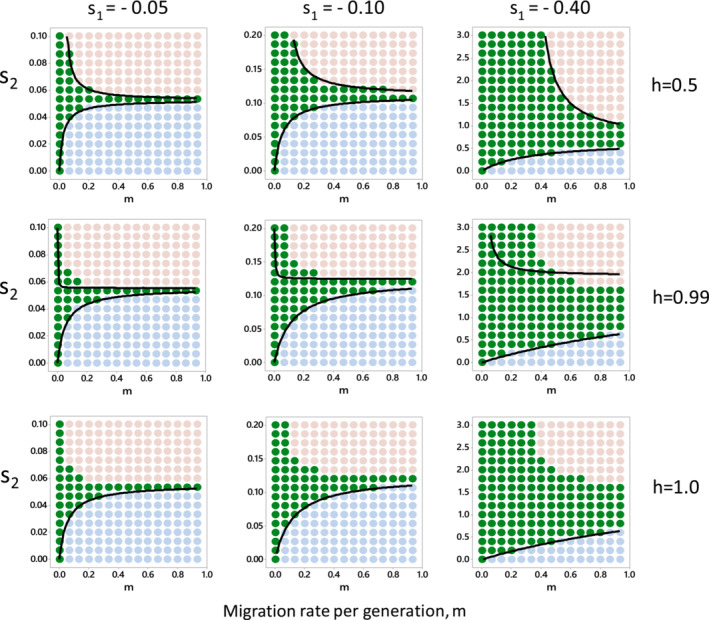
The position of the polymorphism set (green points) in m‐s_2_ space for specified values of h and s_1_. m is the migration rate between the two niches, here assumed the same in both directions; s_1_ and s_2_ represents the fitness advantage of the QQ genotype in niches 1 and 2 respectively; and h represents the level of dominance of Q as specified in Table 1. Points indicate where simulations were run, colors indicate evolutionary outcomes as follows: 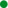
=polymorphism; 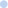
=P wins; 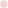
=Q wins. Black curves represent Bulmer boundaries plotted using equations 7. Upper Bulmer boundaries cannot be plotted when h = 1 (bottom row) because of a division by zero in equation 7b. Plots in the first column are for s_1_ = −0.05, in the middle column for s_1_ = −0.10, and in the last column for s_1_ = −0.40. Note variation of y‐axis scale between columns. The top row is for h = 0.5, the middle row h = 0.99, and the bottom row h = 1. The equilibrium frequencies of P in niches 1 and 2 are given in Supplementary Materials.

Equations 7 can be used to locate the positions of the Bulmer boundaries in m‐s_2_ space. The positions of the boundaries are shown as black curves in Figure 2 for specified values of h and s_1_; here for simplicity we set m_12_ = m_21_ = m. Upper boundaries cannot be shown for the case that Q is dominant (h = 1, bottom row) because the equivalents of the two equations from 5a and 5b for p = 1 are then, in fact, the same equation leading to a division by 0 in the formula for s_2_ (equation 7b). Because of the inevitably slow invasion of the population by a recessive allele, the stability of the equilibrium at p = 1 when Q is dominant depends crucially on the quadratic terms and higher order terms in e_1_ and e_2_ that are ignored in deriving equations 5. The approximations in the recurrence equations are less important when the dominance is not complete.

### 2.2 Position of the polymorphism set by simulation of evolutionary outcomes

Evolutionary outcomes were obtained from deterministic simulation of the evolutionary process for specified values of h, m, s_1_ and s_2_, as shown in Figure 2. To obtain evolutionary outcomes, equations 3 and 4 were combined to give equations relating allele frequencies in one generation to their frequencies in the preceding generation. The resulting equations were used to simulate the evolutionary process from an initial frequency of Q of 0.01 in niche 2, for 20,000 generations for s_1_ = −0.05 where evolution is slow, and 10,000 generations for s_1_ = −0.10 or −0.40. Plots of allele frequencies against time revealed that equilibrium was generally reached by the end of each simulation, but in a few cases evolution was still progressing. Because of the time taken to run these simulations it was not practicable to extend the durations of simulations and so an algorithm was used to consider whether or not fixation would be reached. The algorithm was as follows: Alleles were considered to have reached fixation if their final frequencies were within 0.05 of 0 or 1 and between the two niches the final frequencies were within 0.001. The adequacy of this algorithm was checked by plotting gene frequency against time for selected cases close to the boundary of the polymorphism set. In three cases, the algorithm was found to have misidentified the evolutionary outcomes, and these were adjusted accordingly.

### 2.3 Results for local adaptation

Sets of migration and selection rates for which polymorphic evolutionary outcomes occur were found by simulation. These polymorphism sets indicate where local adaptation can exist, and are shown, together with Bulmer boundaries, in Figure 2 for values of h, m, s_1_ and s_2_ chosen to give an idea of the extent of the polymorphism set in four‐dimensional (h, m, s_1_, s_2_) space. Consideration of Table 1 shows that the polymorphism set is symmetrical about h = 0.5, so its extent below mirrors its extent above h = 0.5.

The points in the panels of Figure 2 show the migration rates and s_2_ values at which simulations were run, and their color indicates the evolutionary outcome. The panels differ in levels of dominance, h, and in the fitness of Q in niche 1, s_1_. The set of polymorphic outcomes, here termed the polymorphism set, is shown as green points and Bulmer boundaries as black curves. Points where P and Q go to fixation are colored blue and pink respectively. The lower Bulmer boundaries accurately delineate the boundaries between the blue and the green points. The upper Bulmer boundaries accurately represent the boundaries between the green and pink points when h = 0.5, but not when 0.99, because of the importance of the ignored quadratic terms and higher order terms in the recurrence equations particularly when dominance is complete or nearly complete as discussed earlier. From our numerical results it appears that Bulmer's equations fail for the upper boundary when h ≠ 0.5.

Within each panel, the blue points depict a region where the P allele goes to fixation because Q's fitness in niche 2, s_2_, is too low to prevent Q alleles being swamped in niche 2 by P alleles migrating from niche 1. Figure 2 shows how Q's fitness in niche 2 has to rise to counteract swamping of the Q allele as migration rate increases: this defines the lower boundary of the polymorphism set. The upper boundary of the polymorphism set is more curvilinear than the lower boundary and marks the threshold above which selection on the Q allele in niche 2 is so strong that Q alleles migrating to niche 1 swamp the P alleles in niche 1, and the Q allele goes to fixation (pink points).

The columns of Figure 2 differ in the strength of selection against Q in niche 1, which is weakest in the left‐hand column (s_1_ = −0.05) and strongest in the right (s_1_ = −0.40). When selection against Q in niche 1 is weak the polymorphic set is small, and for polymorphism at higher migration rates, the value of s_2_ has to be tightly matched to the value of s_1_. When selection against Q in niche 1 is higher the polymorphic set increases in size. For example, the range of values of s_2_ at which polymorphism is expected at high migration rates is 0.7 < s_2_ < 1.6 when s_1_ = −0.40 and h = 0.99.

The rows of Figure 2 indicate the effect of dominance, h. Overall the level of dominance does not greatly affect the boundaries of the polymorphism set.

This section has revealed the migration rates and selection strengths that allow the existence of local adaptation (i.e., migration‐selection balance). In the next section, we extend the model of Sibly et al. (2019) to include intermediate levels of dominance, and use it to show that local adaptation always leads to speciation if a new neutral mating trait arises by mutation.

## 3 SECTION 2. LOCAL ADAPTATION LEADS TO SPECIATION IF A NEW NEUTRAL MATING TRAIT ARISES BY MUTATION

To see if, given a suitable mechanism for phenotype matching, local adaptation leads to speciation if a new neutral mating trait arises by mutation, we ran computer simulations of a model developed from Sibly et al. (2019) and applied it to the cases of local adaptation shown in Figure 2. In the model of Sibly et al. (2019), one of the local adaptation alleles is dominant to the other, but now we need to accommodate intermediate dominance as shown in Table 1.

### 3.1 Methods

The model of local adaptation shown in Figure 1 and Table 1 consists of a single locus with three genotypes, PP, PQ and QQ. We now wish to extend the model to include a second locus, independent of and not linked to the PQ locus, with two neutral alleles C and D coding for a mating trait such as plumage color. C is assumed dominant to D. Carriers of C are assumed to mate with other carriers of C with probability α but otherwise at random with probability (1 − α). Similarly DD individuals mate with other DD individuals with probability α but otherwise at random with probability (1 − α). There are three possible genotypes at the CD locus, so considering the two loci there are nine possible genotypes: CCQQ, CCQP, CCPP; CDQQ, CDQP, CDPP; DDQQ, DDQP and DDPP. We write their frequencies r, s, t, u, v, w, x, y and z respectively.

We now derive the recurrence equations showing how the frequencies of the genotypes change under viability selection at the PQ locus. Following the approach of Sibly et al. (2019) we begin by calculating the relative frequencies of offspring genotypes for all possible genotype crosses in an isolated niche with α = 1. These frequencies are shown in Table 2. The first column gives the genotypes and frequencies of fathers, those of mothers are in the top row. The table is symmetrical.

**TABLE 2:** Relative frequencies of the genotypes of the surviving offspring of all possible genotype crosses in an isolated niche for the case α = 1. A = r + s + t + u + v + w, B = x + y + z, f = 1 + s, g = 1 + hs

	CCQQ, *r*	CCQP, *s*	CCPP, *t*	CDQQ, *u*	CDQP, *v*	CDPP, *w*	DDQQ, *x*	DDQP, *y*	DDPP, *z*
CCQQ, *r*	*f* ^*2*^ *r* ^*2*^ */A*	*fgrs/A*	*frt/ A*	*f* ^*2*^ *ru/A*	*fgrv/A*	*frw/A*	0	0	0
CCQP, *s*	*fgrs/A*	*g* ^*2*^ *s* ^*2*^ */A*	*gst/A*	*fgsu/A*	*g* ^*2*^ *sv/A*	*gsw/A*	0	0	0
CCPP, *t*	*frt/A*	*gst/A*	*t* ^*2*^ */A*	*ftu/A*	*gtv/A*	*tw/A*	0	0	0
CDQQ, *u*	*f* ^*2*^ *ru/A*	*fgsu/A*	*ftu/A*	*f* ^*2*^ *u* ^*2*^ */A*	*fguv/A*	*fuw/A*	0	0	0
CDQP, *v*	*fgrv/A*	*g* ^*2*^ *sv/A*	*gtv/A*	*fguv/A*	*g* ^*2*^ *v* ^*2*^ */A*	*gvw/A*	0	0	0
CDPP, *w*	*frw/A*	*gsw/A*	*tw/A*	*fuw/A*	*gvw/A*	*w* ^*2*^ */A*	0	0	0
DDQQ, *x*	0	0	0	0	0	0	*f* ^*2*^ *x* ^*2*^ */B*	*fgxy/B*	*fxz/B*
DDQP, *y*	0	0	0	0	0	0	*fgxy/B*	*g* ^*2*^ *y* ^*2*^ */B*	*gyz/B*
DDPP, *z*	0	0	0	0	0	0	*fxz/B*	*gyz/B*	*z* ^*2*^ */B*

The frequencies in the next generation are obtained from Table 2. In an isolated niche, the frequency of offspring of the CCQQ genotype at the end of the next generation is:

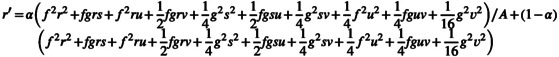

Similarly the frequencies of the other genotypes in the next generation are:

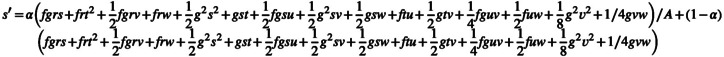




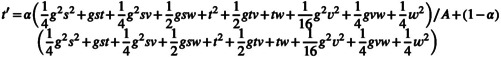




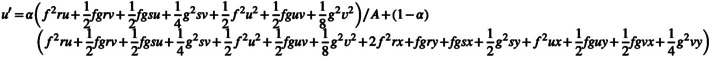




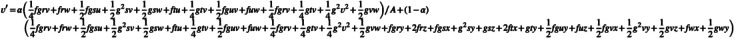




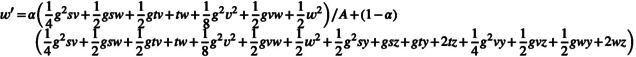




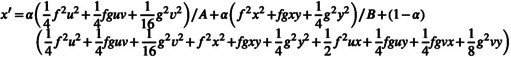




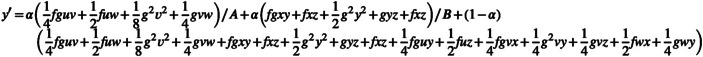




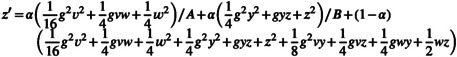

These equations correct errors in (Sibly et al., 2019) for the case α<1. The equations apply to an isolated niche, but need modification if some individuals migrate between niches. Let the population sizes in niches 1 and 2 be M_1_ and M_2_ respectively. We use this notation to allow for later applications to finite populations but in the current infinite population model it is only the ratio M_1_/M_2_ that is important. Let the proportion of individuals in niche 1 emigrating to niche 2 each generation after viability selection be m_12_, and the proportion of those in niche 2 emigrating to niche 1 be m_21_. If the frequency of offspring of genotype i produced in niches 1 and 2 are N_1i_ and N_2i_ respectively, then after migration the number in niche 1 is M_1_N_1i_(1 − m_12_) + M_2_N_2i_m_21_. An analogous formula applies to niche 2, and the number after migration is M_2_N_2i_(1 − m_21_) + M_1_N_1i_m_12_. Assuming the numbers moving in the two directions are equal, then M_1_m_12_ = M_2_m_21_.

This model was run by computer simulation as described below. The computer code used to run the simulations is available with explanatory annotations in Supplementary Materials.

### 3.2 Results for speciation

To see if, given a suitable mechanism for phenotype matching, local adaptation leads to speciation if a new neutral mating trait arises by mutation, we ran the model by computer simulation for each of the cases of local adaptation shown in Figure 2. In each case, we started the simulation with the frequencies, found in Section 1, of the P and Q alleles in niches 1 and 2 under local adaptation. A C allele was introduced at low frequency (1%) into niche 2 as a CDQQ genotype and was found to spread rapidly, and in each case the population speciated into C homozygotes and D homozygotes, with CCQQ predominating in niche 2, and DDPP in niche 1. Eventually all other genotypes are lost. C spreads in niche 2 because its carriers do not mate with most of the incomers from niche 1, who are predominately P‐carriers, so the population of C‐carriers has fewer disadvantageous PP genotypes than the rest of the population in niche 2. Conversely the C‐allele incomers migrating into niche 1 from niche 2 carry disproportionately fewer PP genotypes which alone are advantageous in niche 1, and they mate only with other C‐carrying individuals. The result is that DDPP genotypes are favored over other genotypes in niche 1. It is noteworthy that speciation occurs in our diploid model despite males and females migrating equally between niches, which prevents speciation in haploid models (Gavrilets, 2004, 2006).

In sum, applying this model to the cases of local adaptation shown in Figure 2, in all cases local adaptation led to speciation.

## 4 DISCUSSION

We begin by comparing our model and predictions with those of Felsenstein (1981). Our model is a Felsenstein “two‐allele mechanism” sensu Butlin et al. (2021). The mechanism comprises the PQ and the CD loci, the latter controlling assortative mating. A key difference between Felsenstein's two‐allele models and ours is that Felsenstein's assume linkage between a locus controlling assortative mating and local adaptation loci, whereas ours does not. In our diploid model, the loci are not close to each other in the genome—a situation allowing much greater evolutionary potential. Whether the population will evolve towards speciation depends in our model as in Felsenstein's on the balance between migration and selection: some selection is necessary. We show the values of migration and selection that allow local adaptation quantitatively in our Figure 2. Note that the CD locus is selectively neutral unless there exists both ecological divergence at the PQ locus, and sexual imprinting. The adaptive advantage of sexual imprinting is that it allows locally adapted populations to speciate.

The study of local adaptation has particular significance for the study of parapatric speciation (see, e.g., Butlin et al., 2012; Gavrilets, 2004; Gavrilets, 2014; Kopp et al., 2018; Sakamoto & Innan, 2020; Tobias et al., 2020). Mechanisms of speciation involve females preferring mates whose ecological trait resembles their own, either because the gene coding for the ecological trait also controls female choice (see, e.g., Kopp et al., 2018), or because the females sexually imprint on a newly‐arisen neutral mating trait at a separate, unlinked locus giving rise to phenotype matching, as in the present model. It is clear speciation will occur if the allele for the ecological trait also controls female choice. The outcome is not so clear if females imprint on a neutral mating trait at an unlinked locus, but our results show local adaptation always led to speciation for the cases in Figure 2. On this basis we conjecture that local adaptation always leads to speciation when females imprint on a neutral mating trait at a locus independent of that conferring local adaptation.

Here we first mapped the conditions under which local adaptation can occur, and then showed that with a mechanism of phenotype matching, local adaptation leads to speciation. We discuss these in turn. The emergence of local adaptations depends on the existence of genetically‐based trade‐offs between performance in different niches. Using recurrence equations showing how genotype frequencies change between generations, we found quantitative conditions for stable polymorphisms based on evolutionary outcomes (green circles in Figure 2). We compared the boundaries of the polymorphism set with those derived by Bulmer (1972) and found that one of Bulmer's equations, despite being well known and widely cited and the starting point of several lines of theoretical investigation, is inaccurate except when the level of dominance is 0.5, and inapplicable if one of the alleles is dominant. Bulmer's results are approximations as indicated in Section 1: Methods, and of course approximations will always become inaccurate at some point. Inspection of Figure 2 suggests that Bulmer's approximations work fairly well if selection is weak. The inaccuracies in Bulmer's equations where selection is stronger may need to be accounted for in lines of theoretical investigation which take Bulmer's equations as a starting point, such as Nagylaki (2009), Tomasini and Peischl (2018) and Yeaman and Otto (2011). Comparison of the rows of Figure 2 reveals that overall the level of dominance does not greatly affect the boundaries of the polymorphism set, though local adaptation is a little more likely at h = 0.5 in the key region where m < 0.5. Surprisingly little is known of the distribution of levels of dominance in wild populations (Billiard et al., 2021). Our results show quantitatively the balance required for stable polymorphism between the selection pressures in the two niches: if one is too much stronger than the other then one of the alleles goes to fixation. Stable polymorphism is readily achieved if migration rates between the niches are low. As migration rates increase there is increased mixing of alleles between niches and the conditions for stable polymorphism become more restricted.

For populations in which local adaptations are established, our results show that a mutation of a neutral mating trait at an independent locus can rapidly enable speciation given a suitable mechanism for phenotype matching. Under phenotype matching, carriers of the C allele mate with other carriers of the C allele with probability α. How close this is to what is achieved by sexual imprinting will depend on the mechanism by which sexual imprinting is achieved, but results are nearly identical if offspring imprint on one of the parents, fathers, say, because this case differs from strict phenotype matching only for the case of DD offspring of CD X CD parents (Sibly et al., 2019). The DD offspring from this cross are unlikely to mate (the probability is 1 − α) because they prefer a C phenotype partner but C phenotypes are unlikely to mate with them. The CDs likely mate with each other so their offspring are all imprinted on the C phenotype. These rules then continue down the generations: C phenotypes generally mate with each other, and if they have DD offspring, the DD offspring are unlikely to mate. DD genotypes generally mate with each other throughout. So there are very few mixed‐phenotype breeding pairs. The breeding scheme is like Table 2 except that DD offspring of CD × CD parents are unlikely to mate. This removes the terms divided by A in the equations for x', y', and z' and so strengthens selection against D. This enables C to spread faster than under strict phenotype matching, but the evolutionary outcome is the same. Future work should model other types of sexual imprinting found in nature, perhaps using some of the methods of Verzijden et al. (2005), investigate the effects of varying α, and population sizes as in Sibly et al. (2019), and take account of costs of choosiness, for example the additional time that may be required to find a suitable mating partner. We hope that our two‐locus infinite‐population model will also provide a starting point for studies that include the effects of recombination and drift. Further work is needed to take account of complexities not included in our simplified approach, using methods such as those of Nagylaki (2009), Servedio and Burger (2020), Tomasini and Peischl (2018) and Yeaman and Otto (2011).

How can our predictions be tested? Testing a quantitative theory of local adaptation requires estimation of migration rates and identification of selective advantages, and genome sequencing makes this possible. In a companion paper, we show how selection coefficients can be calculated for large populations in equilibrium in two niches, from allele frequencies, migration rates, population sizes and levels of dominance (Sibly & Curnow, submitted). Migration rates can sometimes be estimated by marking individuals or using genetic markers (e.g., Sunde et al., 2020), and allele frequencies are routinely measured in genomic studies. Levels of dominance are not often measured but as shown here they generally have only small effects.

Our model of speciation makes four predictions. According to the model, whole genome sequences of nascent species should be distinguished by:

1. Locally adaptive genes.
2. Plumage colors coded by single genes which are not linked, i.e., not close in the genome, to the adaptive genes.
3. Despite the lack of linkage a genetic correlation is expected between locally adaptive and plumage color genes.
4. We would expect to find that one of the populations—the one that diverged—went through a bottleneck at the time of divergence. We expect this because in the first generation after a plumage mutation sibs mate with sibs, and inbreeding also occurs in the following generations.

Reviewing studies of genome sequencing, Wolf and Ellegren  (2017) reported that the process generating peaks of elevated genetic differentiation has sometimes been ascribed to divergent selection on individual genes in the face of gene flow between species. We now consider some additional examples. Islands of high divergence, within a genome‐wide background of low divergence have been reported in speckled teal (*Anas flavirostris*) in South America which occur in high and low altitude populations (Graham et al., 2018). Several locally adaptive genes, with F_ST_ values in the range 0.44–0.77, were identified and their functions established. Islands of high divergence, within a genome‐wide background of low divergence, have also been reported in species of Darwin's finches differing in beak size and shape (Han et al., 2017), in four species of Lake Malawi cichlids varying in their adaptations to deepwater environments (Hahn et al., 2017), in two East Asian plovers (Wang et al., 2019) two North American parulid warblers (Toews et al., 2016), three species of sympatric snowfinch (She et al., 2021), within capuchin monkeys comparing those from tropical dry forests with those from lowland rain forest (Orkin et al., 2021), and within treefrogs *Boana platanera* in habitats varying from warm lowland valleys to cooler moist upland forest in the Andes (Medina et al., 2021). These studies demonstrate the potential for genome sequencing to investigate cases of incipient or complete parapatric speciation. If speciation followed the route described here then the loci differentiating two species would be expected to be of two types: (1) genes conferring habitat‐specific adaptions, as in de Leon et al. (2010), Graham et al. (2018), Hahn et al. (2017), Han et al. (2017), Hendry et al. (2009), Medina et al. (2021) and Orkin et al. (2021); (2) genes responsible for the traits used in mate choice, such as plumage color and pattern, as in Toews et al. (2016), or song.

Our results show that given sexual imprinting and local adaptation, a mutation giving males a new mating cue such as a new plumage colour can rapidly spread through a population and in so doing lead to speciation. This could explain some of the diversity of plumage colours seen in birds and cichlids, for example. This idea contrasts with the widely held view that speciation in birds generally follows a period of allopatry.

## AUTHOR CONTRIBUTIONS

**Richard Sibly:** Conceptualization (lead); formal analysis (equal); investigation (lead); methodology (lead); visualization (lead); writing – original draft (lead); writing – review and editing (equal). **Robert Curnow:** Formal analysis (equal); investigation (supporting); methodology (supporting); writing – original draft (supporting); writing – review and editing (supporting).

## CONFLICT OF INTEREST

There are none.

## Supporting information


**Appendix S1** Supporting Information

**Appendix S2** Supporting Information

## Data Availability

The data that supports the findings of this study are available in the supplementary material of this article.
