# Supplementary material for: Sexual imprinting leads to speciation in locally adapted populations
Source: Ecol Evol. 2022 Nov 8;12(11):e9479. doi: 10.1002/ece3.9479 (PMC9643133; doi:10.1002/ece3.9479)
Supplement: Supplementary file 1 — Appendix S1 Supporting Information [file ECE3-12-e9479-s002.docx]

**Supplementary material: Annotated computer code**

The generations are held in the rows of a Minitab worksheet and the frequencies of the genotypes in its columns. Columns c1-c9 hold the frequencies of the 9 genotypes in niche 2 in the order they appear in Table 1, i.e., CCQQ, CCQP … DDPP. Columns c21-c29 hold the frequencies of the 9 genotypes in niche 1. Initial frequencies in generation 1, i.e., in row 1 of the worksheet, were calculated from the equilibrium frequencies of P in niches 1 and 2 as given in supplementary materials. Computer code to implement the recurrence equations takes the relative frequencies in one generation (i.e., in one row of the worksheet) and computes the frequencies in the next. The code for the case $\alpha=1$ is written as a Minitab macro shown in Fig. S1 below and was run in Minitab 18. In the code:

K18 is an index specifying the number of the current generation, k19 is the number of the next generation. K18 is initially set to 0 because it increments by 1 at the start of the macro.

K11 specifies f_2_=1+s_2_, i.e., the fitness of the QQ genotype in niche 2.

K911 specifies f_1_=1+s_1_, i.e., the fitness of the QQ genotype in niche 1.

K14 specifies m_21_, i.e., the proportion of individuals in niche 2 that migrate to niche 1 each generation.

K15 specifies m_12_, i.e., the proportion of individuals in niche 1 that migrate to niche 2 each generation.

K30 specifies g_2_=1+hs_2_, i.e., the fitness of the PQ genotype in niche 2.

K40 specifies f_2_g_2_.

K930 specifies g_1_=1+hs_1_, i.e., the fitness of the PQ genotype in niche 1.

K940 specifies f_1_g_1_.

K51 specifies M_1_, the population size in niche 1

K52 specifies M_2_, the population size in niche 2

The macro first applies the recurrence equations to the genotype frequencies in niche 2 held in columns c1 – c9 of the worksheet, and then to the genotype frequencies in niche 1 held in columns c21 – c29. The genotype frequencies are then normalised in each niche (to represent population regulation) by dividing each genotype frequency by the sum of the frequencies of genotypes in its niche. Finally offspring migrate between niches at rates specified by K14 and K15. (The last two steps occur in opposite order in (Sibly et al., 2019)).

Fig. S1. The Minitab macro implementing the recurrence equations.

let k18=k18+1

let k19=k18+1

let k1=c1(k18)

let k2=c2(k18)

let k3=c3(k18)

let k4=c4(k18)

Initial genotype frequencies in niche 2 transferred to constants k1 – k9

let k5=c5(k18)

let k6=c6(k18)

let k7=c7(k18)

let k8=c8(k18)

let k9=c9(k18)

let k12=k1+k2+k3+k4+k5+k6

A and B for niche 2

let k13=k7+k8+k9

Let k21= k11^2*k1^2+k40*k1*k2+k11^2*k1*k4+k40*k1*k5/2

let k21=k21+k30^2*k2^2/4+k40*k2*k4/2+k30^2*k2*k5/4

Let k21=k21+k11^2*k4^2/4+k40*k4*k5/4+k30^2*k5^2/16

let k21=k21/k12

Let k22=k40*k1*k2+k11*k1*k3*2+k40*k1*k5/2+k11*k1*k6+k30^2*k2^2/2+k30*k2*k3

let k22=k22+k40*k2*k4/2+k30^2*k2*k5/2+k30*k2*k6/2

let k22=k22+k11*k3*k4+k30*k3*k5/2+k40*k4*k5/4

let k22=k22+k11*k4*k6/2

let k22= k22+k30^2*k5^2/8+k30*k5*k6/4

let k22=k22/k12

Let k23=k30^2*k2^2/4+k30*k2*k3+k30^2*k2*k5/4+k30*k2*k6/2

let k23=k23+ k3^2+k30*k3*k5/2+k3*k6+k30^2*k5^2/16+k30*k5*k6/4

let k23=k23+k6^2/4

let k23=k23/k12

Let k24=k11^2*k1*k4+k40*k1*k5/2+k40*k2*k4/2+k30^2*k2*k5/4+k11^2*k4^2/2

Recurrence equations applied to niche 2

Let k24=(k24+k40*k4*k5/2+k30^2*k5^2/8)/k12

Let k25=k40*k1*k5/4+k11*k1*k6+k40*k2*k4/2+k30^2*k2*k5/2+k30*k2*k6/2+k11*k3*k4+k30*k3*k5/4

Let k25=k25+k40*k4*k5/2+k11*k4*k6+k40*k1*k5/4+k30*k3*k5/4+k30^2*k5^2/4+k30*k5*k6/2

let k25=k25/k12

Let k26=k30^2*k2*k5/4+k30*k2*k6/2+k30*k3*k5/2+k3*k6

Let k26=(k26+k30^2*k5^2/8+k30*k5*k6/2+k6^2/2)/k12

Let k27=(k11^2*k4^2/4+k40*k4*k5/4+k30^2*k5^2/16)/k12

Let k27=k27+(k11^2*k7^2+k40*k7*k8+k30^2*k8^2/4)/k13

Let k28=(k40*k4*k5/4+k11*k4*k6/2+k30^2*k5^2/8+k30*k5*k6/4)/k12

Let k28=k28+(k40*k7*k8+k11*k7*k9+k30^2*k8^2/2+k30*k8*k9+k11*k7*k9)/k13

Let k29=(k30^2*k5^2/16+k30*k5*k6/4+k6^2/4)/k12 +(k30^2*k8^2/4+k30*k8*k9+k9^2)/k13

let k91=c21(k18)

let k92=c22(k18)

let k93=c23(k18)

let k94=c24(k18)

Initial genotype frequencies in niche 1 transferred to constants k91 – k99

let k95=c25(k18)

let k96=c26(k18)

let k97=c27(k18)

let k98=c28(k18)

let k99=c29(k18)

let k912=k91+k92+k93+k94+k95+k96

A and B for niche 1

let k913=k97+k98+k99

Let k31= k911^2*k91^2+k940*k91*k92+k911^2*k91*k94+k940*k91*k95/2

let k31=k31+k930^2*k92^2/4+k940*k92*k94/2+k930^2*k92*k95/4

Let k31=k31+k911^2*k94^2/4+k940*k94*k95/4+k930^2*k95^2/16

let k31=k31/k912

Let k32=k940*k91*k92+k911*k91*k93*2+k940*k91*k95/2+k911*k91*k96+k930^2*k92^2/2+k930*k92*k93

let k32=k32+k940*k92*k94/2+k930^2*k92*k95/2+k930*k92*k96/2

let k32=k32+k911*k93*k94+k930*k93*k95/2+k940*k94*k95/4

let k32=k32+k911*k94*k96/2

let k32= k32+k930^2*k95^2/8+k930*k95*k96/4

let k32=k32/k912

Let k33=k930^2*k92^2/4+k930*k92*k93+k930^2*k92*k95/4+k930*k92*k96/2

let k33=k33+ k93^2+k930*k93*k95/2+k93*k96+k930^2*k95^2/16+k930*k95*k96/4

let k33=k33+k96^2/4

let k33=k33/k912

Let k34=k911^2*k91*k94+k940*k91*k95/2+k940*k92*k94/2+k930^2*k92*k95/4+k911^2*k94^2/2

Let k34=(k34+k940*k94*k95/2+k930^2*k95^2/8)/k912

Recurrence equations applied to niche 1

Let k35=k940*k91*k95/4+k911*k91*k96+k940*k92*k94/2+k930^2*k92*k95/2+k930*k92*k96/2+k911*k93*k94+k930*k93*k95/4

Let k35=k35+k940*k94*k95/2+k911*k94*k96+k940*k91*k95/4+k930*k93*k95/4+k930^2*k95^2/4+k930*k95*k96/2

let k35=k35/k912

Let k36=k930^2*k92*k95/4+k930*k92*k96/2+k930*k93*k95/2+k93*k96

Let k36=(k36+k930^2*k95^2/8+k930*k95*k96/2+k96^2/2)/k912

Let k37=(k911^2*k94^2/4+k940*k94*k95/4+k930^2*k95^2/16)/k912

Let k37=k37+(k911^2*k97^2+k940*k97*k98+k930^2*k98^2/4)/k913

Let k38=(k940*k94*k95/4+k911*k94*k96/2+k930^2*k95^2/8+k930*k95*k96/4)/k912

Let k38=k38+(k940*k97*k98+k911*k97*k99+k930^2*k98^2/2+k930*k98*k99+k911*k97*k99)/k913

Let k39=(k930^2*k95^2/16+k930*k95*k96/4+k96^2/4)/k912 +(k930^2*k98^2/4+k930*k98*k99+k99^2)/k913

let k101=k21+k22+k23+k24+k25+k26+k27+k28+k29

let k21=k21/k101

let k22=k22/k101

let k23=k23/k101

let k24 =k24/k101

Normalise frequencies in niche 2 so they sum to 1

let k25 =k25/k101

let k26 =k26/k101

let k27 =k27/k101

let k28 =k28/k101

let k29 =k29/k101

let k102=k31+k32+k33+k34+k35+k36+k37+k38+k39

let k31 =k31/k102

let k32 =k32/k102

let k33 =k33/k102

let k34 =k34/k102

Normalise frequencies in niche 1 so they again sum to 1

let k35 =k35/k102

let k36 =k36/k102

let k37 =k37/k102

let k38 =k38/k102

let k39 =k39/k102

let c1(k19)= k52*(1-k14)*k21+ k51*k15*k31

let c2(k19)= k52*(1-k14)*k22+ k51*k15*k32

let c3(k19)= k52*(1-k14)*k23+ k51*k15*k33

let c4(k19)= k52*(1-k14)*k24+ k51*k15*k34

Frequencies in niche 2 after some offspring have migrated between niches

let c5(k19)= k52*(1-k14)*k25+ k51*k15*k35

let c6(k19)= k52*(1-k14)*k26+ k51*k15*k36

let c7(k19)= k52*(1-k14)*k27+ k51*k15*k37

let c8(k19)= k52*(1-k14)*k28+ k51*k15*k38

let c9(k19)= k52*(1-k14)*k29+ k51*k15*k39

let c21(k19)= k52*k14*k21+ k51*(1-k15)*k31

let c22(k19)= k52*k14*k22+ k51*(1-k15)*k32

let c23(k19)= k52*k14*k23+ k51*(1-k15)*k33

let c24(k19)= k52*k14*k24+ k51*(1-k15)*k34

Frequencies in niche 1 after some offspring have migrated between niches

let c25(k19)= k52*k14*k25+ k51*(1-k15)*k35

let c26(k19)= k52*k14*k26+ k51*(1-k15)*k36

let c27(k19)= k52*k14*k27+ k51*(1-k15)*k37

let c28(k19)= k52*k14*k28+ k51*(1-k15)*k38

let c29(k19)= k52*k14*k29+ k51*(1-k15)*k39

let c30(k19)=k19

Sibly, R. M., Pagel, M., Curnow, R. N., Edwards, J., 2019. How phenotypic matching based on neutral mating cues enables speciation in locally adapted populations. Ecology and Evolution 9, 13506-13514, doi:10.1002/ece3.5806.
